# Bacterial communities of *Aphis gossypii* and *Myzus persicae* (Hemiptera: Aphididae) from pepper crops (*Capsicum* sp.)

**DOI:** 10.1038/s41598-019-42232-8

**Published:** 2019-04-08

**Authors:** Jenny Johana Gallo-Franco, Diana Nataly Duque-Gamboa, Nelson Toro-Perea

**Affiliations:** 1 Biology department (Departamento de biología), Universidad del Valle, Street 13 No. 100-00, 760032 Cali, Colombia; 2 Centre for Bioinformatics and Photonics-CIBioFi, Universidad del Valle, Street 13 # 100-00, Building 320 No. 1069, 760032 Cali, Colombia

## Abstract

Insects harbor a wide variety of microorganisms that form complex and changing communities and play an important role in the biology and evolution of their hosts. Aphids have been used as model organisms to study microorganism-insect interactions. Almost all aphids are infected with the obligate endosymbiont *Buchnera aphidicola* and can host different bacteria that allow them to acquire traits of agronomic importance, such as resistance to high temperatures and/or defense against natural enemies. However, the bacterial communities of most aphid species remain poorly characterized. In this study, we used high-throughput DNA sequencing to characterize the bacterial communities of *Aphis gossypii* and *Myzus persicae* from two cultivable pepper species, *Capsicum frutescens* (Tabasco variety) and *C*. *annuum* (Cayenne variety), in four localities of southwestern Colombia. In addition, we evaluated the dynamics of *A*. *gossypii*-associated microorganisms on a seasonal basis. Our results show that the bacterial communities of *A*. *gossypii* and *M*. *persicae* are dominated by the primary endosymbiont *B*. *aphidicola*, while the presence of the facultative symbiont *Arsenophonus* sp. was only detected in one *A*. *gossypii* population from cayenne pepper. In addition to these two known symbionts, eight bacterial OTUs were identified that presented a frequency of 1% or more in at least one of the analyzed populations. The results show that the bacterial communities of aphids associated with pepper crops appears to be structured according to the host aphid species and the geographical location, while no differences were observed in the diversity of bacteria between host plants. Finally, the diversity and abundance of the *A*. *gossypii* bacterial community was variable among the four sampling points evaluated over the year and showed a relation with the aphid’s population dynamics. This study represents the first approach to the knowledge of the bacterial community present in chili pepper aphids from Colombia. Nevertheless, more in-depth studies, including replicates, are required to confirm the patterns observed in the microbial communities of aphids from pepper crops.

## Introduction

Insects are associated with various microorganisms, many of which are capable of significantly affecting different aspects of their biology^1^. Through long periods of co-evolution, microorganisms and insects have developed complex interactions, ranging from pathogenesis to an obligate mutualism^2^. Although a great deal of attention has been devoted to pathogenic bacteria, it is likely that most insect species harbor a microbial community that even outnumbers their own cells, which mainly consists of non-pathogenic and free-living bacteria^3^.

Aphids (Hemiptera: Aphididae) are a diversified group of specialized insects that feed on the sap of plants and have been recognized as model organisms for studying microorganism-insect relationships^4^. Almost all aphids present a primary endosymbiont, *Buchnera aphidicola*, which is transmitted in a stable manner from mother to offspring and is essential to compensate for the nutritional deficiencies of these sap-sucking insects^5^. This endosymbiont has been shown to have a co-speciation pattern with aphids, the result of an ancestral infection and its vertical transmission along the host lineage^5^.

In addition to *Buchnera*, aphids can present a series of secondary or facultative symbionts that are inherited vertically but can also be transferred horizontally within and between host species^4^. Although these symbionts are not necessary for the survival of the host, they appear to replicate only in association with the host and can confer characteristics of agronomic, ecological and evolutionary importance to insects under certain environmental conditions^4^. For example, these bacteria can increase host resistance to pathogens and natural enemies^6^. The presence of certain species of bacteria seems to confer resistance to high temperatures, which could affect the range and variability of climates that a host organism tolerates and determine its range of geographical distribution^7^. These microorganisms can also be involved in determining the host plant that is attacked by aphids^8^ and can facilitate the transmission of viruses^9–11^, an effect of the symbiont-insect interaction that is of great relevance for agronomic management due to the role that aphids can play as virus vectors for different host plants^12,13^. The ability of facultative symbionts to introduce new hereditary traits in their hosts can affect both the abundance of symbionts and the success and evolution of aphids^4^.

Several molecular characterization studies have assessed the diversity of microorganisms in the pea aphid, *Acyrthosiphon pisum*, focusing on a limited range of symbionts in geographical regions such as Europe, Japan and the United States^14–19^. Studying the diversity of microorganisms based only on the search for known symbionts, limits our knowledge about the possible interactions between members of microbial communities and limits the possibility of finding new symbionts^20^, especially in non-model aphid species. High-throughput sequencing technologies are now replacing traditional PCR for the investigation of microbial communities^21^. These sequencing technologies have become an efficient tool to characterize the diversity and structure of bacterial communities in aphids because they do not rely on prior knowledge about the diversity of the communities investigated, allowing for a better understanding of the roles of microorganisms in these insects and the importance of these symbiotic associations^18,22–25^, specially in non-model aphids^26–28^.

The cotton aphid, *Aphis gossypii* (Glover 1877), is considered one of the most destructive aphid species in the world^29^. Although this insect has a cosmopolitan distribution, it is particularly abundant in the tropics and attacks at least 64 species of plants of economic importance, primarily of the families Cucurbitaceae, Malvaceae and Solanaceae^30^. *Myzus persicae* (Sulzer 1776), known as the green peach aphid, is a cosmopolitan aphid that infests several plants of economic importance, primarily dicots. It has been reported that this aphid can transmit more than 100 viral diseases to approximately 30 different plant families^31^. Severe infestations of *A*. *gossypii* and *M*. *persicae* in crops of commercial interest can cause chlorosis, necrosis, wilting, stunted growth, flower and fruit abortion, leaf distortion and defoliation, among other symptoms^32^. These two aphid species, especially *A*. *gossypii*, are considered representative pests of pepper crops^29^, causing several different production constraints^32^. The cultivation of this Solanaceae is extremely important to the horticultural production of Colombia, because its growth rate is higher than the average of other crops, with a 13% rate per year^33^. Three varieties of pepper, *Capsicum frutescens* (Tabasco var.), *C*. *annuum* (Cayenne var.) and *C*. *chinense* (Habanero var.), are the most cultivated for domestic consumption and for export purposes.

Despite the economic importance of *A*. *gossypii* and *M*. *persicae* worldwide, the diversity of their microorganism communities has remained poorly characterized, and no studies have identified factors that may affect these communities. In this study, we performed mass sequencing of the 16S rRNA gene using an Illumina platform to evaluate the effects of the aphid species, host plant, geographical location and the time of year in the structuring of bacterial communities in aphids associated with pepper crops in the Colombian southwest.

## Results

### Field sampling

**Figure 1:**
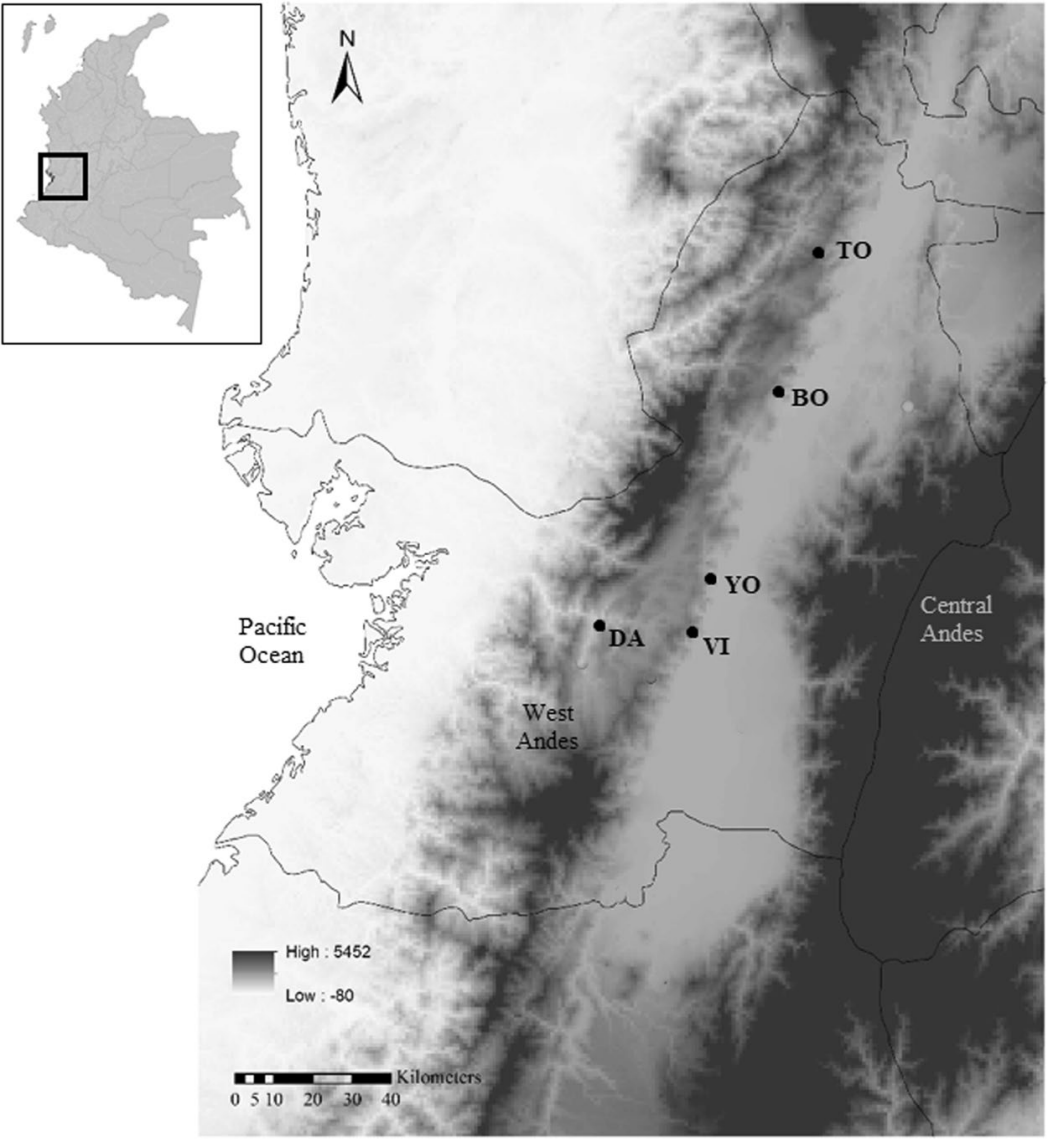
Sample locations for *A*. *gossypii* and *M*. *persicae* in southwestern Colombia. Toro (TO), Bolivar (BO), Yotoco (YO), Vijes (VI), Dagua (DA). The map was prepared using ArcGIS 10.3.

Twelve aphid samples, each consisting of a pool of 15 individuals, collected from crops of *C*. *frutescens* (Tabasco var.) and *C*. *annum* (Cayenne var.) in five localities of southwestern Colombia, were subjected to 16s rRNA Illumina Miseq sequencing, covering variable regions V3 and V4 (Table 1, Fig. 1). Eight samples of *A*. *gossypii* and *M*. *persicae* were used to compare diversity among localities, aphid species and host plants, and four samples collected in the Yotoco locality were used to evaluate seasonal dynamics of the *A*. *gossypii* bacterial community.

**Table 1:** Aphid sampling locations for pepper crops in southwestern Colombia. In the sample code, the first two letters correspond to the aphid species, the third letter corresponds to the locality and the number refers to the plant variety, 1 for Tabasco and 2 for Cayenne.

Location	Latitude/Longitude	Sample code	Collection date	Aphid species	Pepper variety
Toro	N 4.60600; W-76.08300	Ag_T1	Feb-2017	*A*. *gossypii*	Tabasco (*C*. *frutescens*)
		Ag_T2	Feb-2017	*A*. *gossypii*	Cayenne (*C*. *annum*)
		Mp_T2	Feb-2017	*M*. *persicae*	Cayenne (*C*. *annum*)
Vijes	N 3.70500; W-76.43000	Ag_V1	Feb-2017	*A*. *gossypii*	Tabasco (*C*. *frutescens*)
		Ag_V2	Feb-2017	*A*. *gossypii*	Cayenne (*C*. *annum*)
Dagua	N 3.75300; W-76.65600	Ag_D1	Feb-2017	*A*. *gossypii*	Tabasco(*C*. *frutescens*)
Bolívar	N 4.30000; W-76.20800	Ag_B2	Feb-2017	*A*. *gossypii*	Cayenne (*C*. *annum*)
		Mp_B2	Feb-2017	*M*. *persicae*	Cayenne (*C*. *annum*)
Yotoco	N 3.82526; W-76.39697	Ag_T1_Sep	Sep-2016	*A*. *gossypii*	Tabasco(*C*. *frutescens*)
		Ag_T1_Dic	Dic-2016	*A*. *gossypii*	Tabasco(*C*. *frutescens*)
		Ag_T1_Mar	Mar-2017	*A*. *gossypii*	Tabasco(*C*. *frutescens*)
		Ag_T1_Jun	Jun-2017	*A*. *gossypii*	Tabasco(*C*. *frutescens*)

### Sequence analysis and taxonomic assignment

After filtering for sequence quality and size, between 15392 and 16721 reads were recovered for eight samples of *A*. *gossypii* and *M*. *persicae* (Table 2). The rarefaction curves tended toward saturation (Fig. S1), and the coverage value of the sequencing data, with a dissimilarity value of 0.03, was greater than 99% in all the samples (Table 2). These results suggest that the depth of sequencing used was adequate to detect most of the bacterial diversity of *A*. *gossypii* and *M*. *persicae* from pepper crops.

**Table 2:** Bacterial diversity indices of *Aphis gossypii* and *Myzus persicae* through sequencing analysis of 16S rRNA gene amplicons. The operational taxonomic units (OTUs) were established at 97% identity in the sequence pairing.

Sample	Host plant	Location	No. reads	Coverage	No. OTUs	Simpson	Shannon	Chao
AgT1	Tabasco	Toro	16418	0.99	175	1.19	0.62	196.0
AgV1	Tabasco	Vijes	16012	0.99	321	1.54	1.30	371.4
AgD1	Tabasco	Dagua	16443	0.99	289	1.83	1.30	350.7
AgT2	Cayenne	Toro	16683	0.99	65	1.13	0.38	69.4
AgV2	Cayenne	Vijes	16591	0.99	284	1.25	0.87	303.1
AgB2	Cayenne	Bolívar	16488	0.99	334	1.35	1.12	352.2
MpT2	Cayenne	Toro	15392	0.99	29	1.01	0.06	74.0
MpB2	Cayenne	Bolívar	16721	0.99	109	1.08	0.30	125.7

The obtained reads were distributed into 454 OTUs with an identity level of 97%. These OTUs included 22 phyla, with Proteobacteria being the most dominant phylum, with a relative abundance of 93.83% (Average values across all samples, Fig. S2). The dominant class of the samples was Gammaproteobacteria with a relative abundance of 91.3% (Fig. S3). At the family level, Enterobacteriaceae was the dominant family, with a relative abundance of 87.4% (Fig. S4). The relative abundances of the representative OTUs of the bacterial communities of *A*. *gossypii* and *M*. *persicae* (>1% relative frequency in at least one sample) are shown in Table 3. The values of relative abundances obtained correspond only to approximate proportions of the bacterial OTUs in each sample evaluated.

**Table 3:** Relative frequencies of representative OTUs (>1% in at least one sample) of the *A. gossypii* and *M*. *persicae* bacterial communities.

Taxon	Relative frequencies (%)							
	Tabasco			Cayenne				
	Ag_T1	Ag_V1	Ag_D1	Ag_T2	Ag_V2	Ag_B2	Mp_T2	Mp_B2
*Buchnera aphidicola*	89.91	86.93	70.27	93.48	78.41	83.71	98.21	95.24
*Pseudomonas* sp.	0.29	0	17.48	1.40	1.82	0	0	0.53
Enterobacteriaceae^b^	0.60	0.33	0.27	0.59	0.40	0.28	1.05	0.40
*Arsenophonus* sp.	0	0	0	2.01	0	0	0	0
ACK-M1^b^	0.03	0.77	0.31	0	1.02	1.08	0	0.05
Pseudomonadaceae^b^	1.87	0	0.96	0.24	3.51	0	0	0.23
Bacteroidetes^a^	0.11	0.87	0.67	0	0.71	1.40	0	0.06
Pelagibacteraceae^b^	0.17	0.72	0.50	0	0.46	0.97	0	0.09
*Selenomonas* sp.	1.05	0.23	0.42	0.31	0.23	0.31	0	0.33
*Sphingomonas* sp.	0.08	0	0.15	0.18	2.47	0	0.09	0.08

^a^Taxonomic assignment to phylum.

^b^Taxonomic assignment to family.

Two known endosymbionts, *B*. *aphidicola* and *Arsenophonus* sp., were observed, of which *B*. *aphidicola* was dominant in the bacterial communities of both aphid species and was observed in all samples, as well as an OTU belonging to an unreported genus of the family Enterobacteriaceae. The highest relative abundance of *B*. *aphidicola* was observed in the MpT2 and MpB2 samples of *M*. *persicae*, and the lowest relative abundance was observed in the AgD1 sample of *A*. *gossypii* (Table 3). In contrast, *Arsenophonus* sp. was only observed in the AgT2 sample, with a relative abundance of 2.01% (Table 3).

### Phylogenetic reconstruction

**Figure 2:**
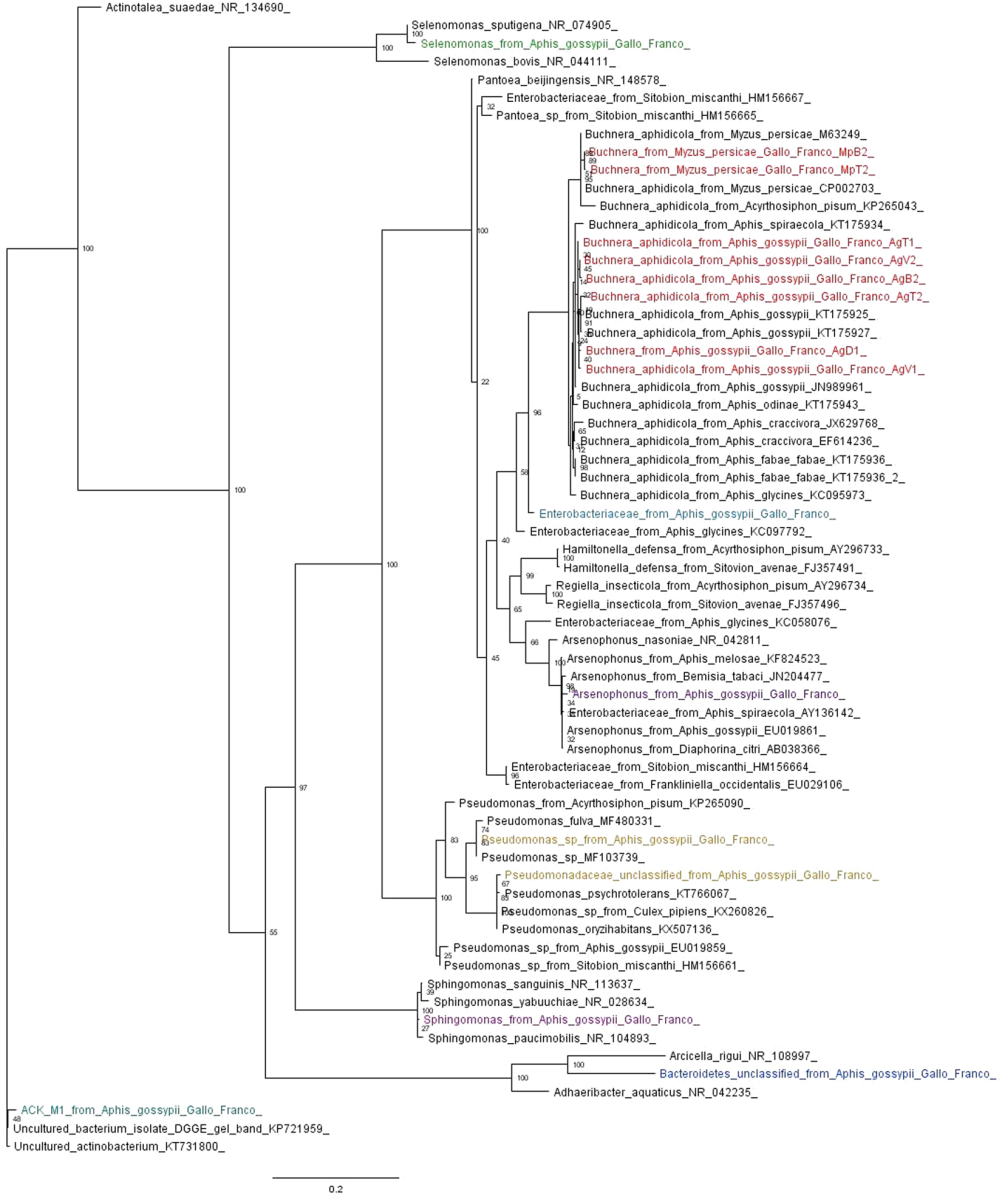
Phylogenetic analysis of the representative bacterial OTUs (>1%) associated with *A*. *gossypii* and *M*. *persicae* from pepper crops (the names of the sequences from our work end in Gallo_Franco and are colored by genus) and related sequences obtained from GenBank (the accession number of each sequence is shown at the end of the name). The phylogenetic inference was made using maximum likelihood and the GTR model.

According to the *Greengenes* database, the taxonomic assignment agrees with the phylogenetic tree produced with 16S rRNA gene sequences obtained in this study and with GenBank sequences (Fig. 2). The clade formed by the primary endosymbiont, *B*. *aphidicola*, was monophyletic, with groups structured according to its host aphid (Fig. 2). *Selenomonas* sp., *Sphingomonas* sp. and *Pseudomonas* sp. grouped into monophyletic clades with bacteria of the same genus (Fig. 2). However, due to the high percentages of similarity (above 97%) with different species of bacteria, a taxonomic resolution at the species level was not achieved. For example, the bacterium *Pseudomonas* sp. reported in our study, with a relative abundance of 17.48% in the locality of Dagua, was grouped with the species *Pseudomonas fulva* (MF480331) and with an unknown species of *Pseudomonas* (MF103739) with 100% similarity (Fig. 2). The secondary endosymbiont *Arsenophonus* sp., reported in the AgT2 sample, formed a monophyletic clade with sequences of *Arsenophonus* sp. reported in other insects, such as *Bemisia tabaci* and *Diaphorina citri* (Fig. 2). The Bacterium belonging to the family Enterobacteriaceae reported in this study was in a clade close to that of *B*. *aphidicola*, as well as a bacterium belonging to the family Enterobacteriaceae reported by GenBank for *Aphis glycines* (KC097792). However, the two bacteria were not associated with any known symbiont (Fig. 2). The genetic distance (p-distance) between the Enterobacteriaceae reported in *A*. *glycines* (KC097792) and the Enterobacteriaceae observed in this study is 0.027. This genetic distance is similar to that observed between the *B*. *aphidicola* sequences of *A*. *gossypii* and *M*. *persicae* reported in this study (0.024–0.026).

### Comparative analysis of bacterial communities in aphids in localities and in host plants

The samples that presented the lowest diversity indices were AgT2 (*A*. *gossypii*) and MpT2 (*M*. *persicae*), both from the Toro locality (Table 2), while the highest diversity indices were observed for the samples from *A*. *gossypii*, AgD1 and AgV1, in the localities of Dagua and Vijes, respectively (Table 2). In those localities (Toro and Vijes) where aphids were collected from the two varieties of pepper, the bacterial community associated with the Tabasco variety was the most diverse according to all measured diversity indices. In the localities of Toro and Bolívar, where both *A*. *gossypii* and *M*. *persicae* were collected from the Cayenne variety, *A*. *gossypii* presented the most diverse bacterial community (Table 2).

**Figure 3:**
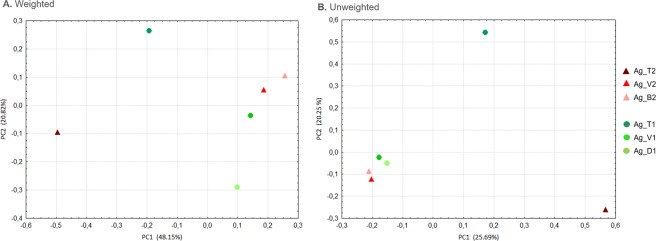
Principal coordinates analysis of the *A*. *gossypii* bacterial communities among host plants. The triangles and circles correspond to samples from Cayenne and Tabasco pepper plants, respectively. The weighted and unweighted UniFrac indices were used to calculate the distances between pairs of samples.

Although the diversity of bacteria seems to be greater in aphids collected from the Tabasco pepper variety (Table 2), the Principal Coordinates Analysis (PCoA), performed to determine the structure of the bacterial communities, did not show a clear clustering by host plant (Fig. 3). In the PCoA, Toro was separated from the other localities, and the Cayenne and Tabasco samples in this locality are distant from each other compared with the other localities (Fig. 3). Equally, the AMOVA (Analysis of Molecular Variance) showed that there are no significant differences in the microbial community among aphids from Cayenne and Tabasco (Fs = 1.306, p = 0.215).

**Figure 4:**
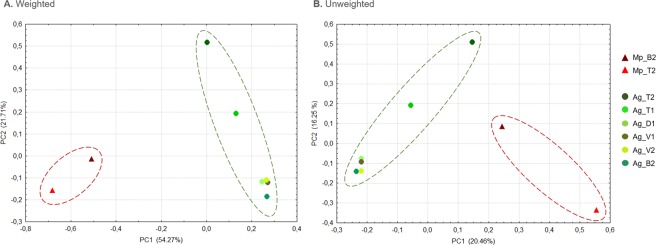
Principal coordinates analysis of the *A. gossypii* (green circles) and *M. persicae* (red triangles) bacterial communities. The weighted and unweighted UniFrac indices were used to calculate the distances between pairs of samples.

The PCoA performed among aphid species showed two groups that differentiated between the *M*. *persicae* and *A*. *gossypii* samples, especially when the weighted UniFrac index was used (Fig. 4). This result shows that the bacterial communities are structured according to the host aphid. A microbial community composition different from the other localities was still observed in the locality of Toro (Fig. 4).

### Seasonal dynamics of the *A*. *gossypii* bacterial community

The temporal dynamics analysis of the *A*. *gossypii* bacterial community was carried out using one sample for each of the four different times point of the year we studied, for which more than 14000 sequences of the 16S rRNA gene were obtained (Table 4). The rarefaction curves tended toward saturation (Fig. S5), and the coverage value of the sequencing data, with a dissimilarity value of 0.03, was above 99% (Table 4), indicating that the sampling of the microbial communities was adequate to identify most of the bacterial diversity of *A*. *gossypii* in the Yotoco experimental plot.

**Table 4:** Seasonal bacterial diversity indices of *A*. *gossypii* sampled four times over the course of a year through 16S rRNA gene sequence analysis. The operational taxonomic units (OTUs) were established at 97% identity in the sequence pairing.

Sample	No. reads	Coverage	No. OTUS	Simpson	Shannon	Chao
Ag_T1_Sep	15234	0.99	49	1.046	0.162	57.75
Ag_T1_Dic	14096	0.99	731	6.447	4.257	738.65
Ag_T1_Mar	15241	0.99	46	1.020	0.087	67.11
Ag_T1_Jun	15206	0.99	26	1.007	0.034	29.27

From the 16S rRNA gene sequences, a maximum of 731 OTUs were identified, with 97% identity. These OTUs were assigned to 24 phyla, among which the Proteobacteria phylum predominated with a relative abundance of 88.4% (Fig. S6) and 61 classes, with the class Gammaproteobacteria being predominant with a relative abundance of 84.9%. We identified 139 families, among which Enterobacteriaceae was the most abundant with a relative abundance of 84.6%. The relative abundances of the bacteria most representative of the bacterial community (>1% of relative frequency in at least one sample) are shown in Table 5. The dominant bacteria in all the samples was the primary endosymbiont *B*. *aphidicola*, with relative frequencies between 38.4 and 98.7%. The lowest relative abundance of *B*. *aphidicola* was observed in the sample taken in December 2016, and the highest relative abundance was observed in June 2017 (Table 5).

**Table 5:** Seasonal relative frequencies of the representative OTUs (>1% in at least one sample) of the bacterial community of *A*. *gossypii* in an experimental plot of Yotoco.

Taxon	Relative frequencies (%)			
	Ag_Sep_16	Ag_Dic_16	Ag_Mar_17	Ag_Jun_17
*Buchnera aphidicola*	96.40	38.38	97.97	98.67
^b^Enterobacteriaceae	1.18	0.16	1.13	1.06
^b^ACK-M1	0	4.36	0	0
^a^Bacteroidetes	0	3.25	0	0
^b^Pelagibacteraceae	0	2.14	0	0
^b^Cerasicoccaceae	0	2.15	0	0
Bacteria	0	1.50	0	0
*Selenomonas* sp.	0	1.09	0	0
^b^Chitinophagaceae	0	2.30	0	0
^b^Comamonadaceae	0	1.94	0	0
*Limnohabitans* sp.	0	1.64	0	0
*Fluviicola* sp.	0	1.59	0	0
*Allobaculum* sp.	0	1.09	0	0
*Rhodococcus* sp.	1.08	0	0	0

^a^Taxonomic assignment to filum.

^b^Taxonomic assignment to family.

**Figure 5:**
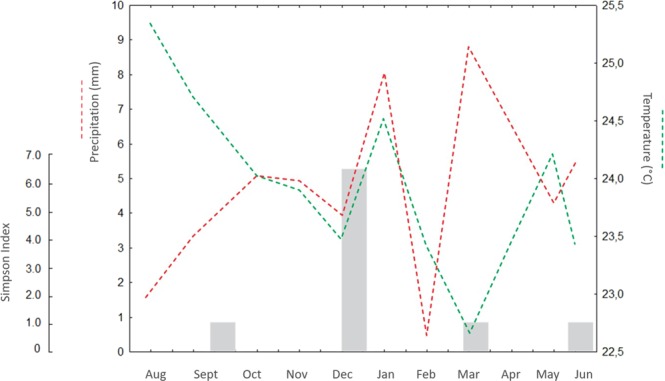
Variation in the diversity of the *Aphis gossypii* bacterial community in four temporal sampling points compared to fluctuations in temperature and precipitation in an experimental plot in the locality of Yotoco (Data taken from the Cenicaña-Yotoco weather station). The gray bars represent the alpha diversity present of the four samplings made throughout the year 2017.

The samples from the month of December presented the highest diversity index and the highest number of bacterial OTUs. The other three samplings presented diversity indices similar to each other but lower than that observed in the sample from December. When we compare this seasonal variation in the *A*. *gossypii* bacterial community with the average rainfall and temperature data in the Yotoco locality, no relationship was observed between bacterial diversity and environmental conditions (Fig. 5).

## Discussion

In this study, the microbial diversity of *A*. *gossypii* and *M*. *persicae* from pepper crops of southwestern Colombia was characterized. These two aphid species are of global economic importance, and little is known regarding their interactions with microorganisms, which have proven to be key elements in the evolution and ecology of insects^4,34^. The results showed that the bacterial communities of *A*. *gossypii* and *M*. *persicae* are dominated by the endosymbiont *B*. *aphidicola*. The dominant presence of the primary endosymbiont in both aphid species is in agreement with the obligate mutualistic relationship that has been reported between these insects and their primary endosymbiont. This relationship is crucial, since aphids are dependent on this microorganism for the production of the essential amino acids, vitamins and sterols that are necessary for their normal development and reproduction^35^. Similarly, aphids provide nutrients for *B*. *aphidicola*, including non-essential amino acids and carbohydrates that are abundant in the aphid diet, which is based on the phloem of plants, or that are produced by the aphids themselves^35–37^.

In addition to the primary endosymbiont *B*. *aphidicola*, we found the secondary endosymbiont *Arsenophonus* sp. in *A*. *gossypii* specimens collected from the Cayenne pepper variety from Toro locality. Former studies have found that natural populations of *A*. *gossypii* can be associated with six genera of secondary or facultative endosymbionts (*Hamiltonella*, *Arsenophonus*, *Regiella*, *Rickettsia*, *Serratia* and *Wolbachia*)^24,38–44^, while natural populations of *M*. *persicae* with three genera of symbionts (*Regiella*, *Serratia* and *Hamiltonella*)^27^. However, despite the biological importance that these endosymbionts may have, they are only partially distributed in the populations of some aphid species and are even completely absent in others^24,26,27^. The absence of different secondary symbionts in our samples of aphids, may be because maintaining a secondary symbiont, despite being a benefit, can infer a fitness cost to the host^45^, which suggests that the persistence of a symbiont is determined by the balance between cost and benefit^46^. Fukatsu *et al*.^47^, proposed a simple model to explain the presence or absence of a symbiont in natural populations, in which the infection frequency of an endosymbiont in a host population is determined mainly by three parameters: fidelity of vertical transmission, fitness effect on the host and frequency of horizontal transmission. These patterns of variation of secondary symbionts in natural populations are consistent with previous studies realized in *A*. *gossypii* populations. Najar-Rodríguez *et al*.^41^, evaluated the bacterial diversity of *A*. *gossypii* in localities of Australia and Japan, and reported the presence of the endosymbiont *Arsenophonus* in some of the evaluated populations. In Chinese populations of *A*. *gossypii*, Zhao *et al*.^24^ report the presence of the symbionts *Arsenophonus* and *Hamiltonella* in all the analyzed populations, nevertheless the other three symbionts were not detected in their study.

Unlike what was believed a few years ago, when *Arsenophonus* was considered an absent endosymbiont in aphids^4,48^, recent studies have shown that it is a bacterium widely distributed in various insect species, including those of the family Aphididae^49^. Jousselin *et al*.^44^, in a study of 86 aphid species, reported an *Arsenophonus* infection incidence of 7%. These researchers reported an especially high incidence of *Arsenophonus* in the *Aphis* genus, more than 31% of the species were infected, but absence of *Arsenophonus* in the *Myzus* genus. Our results confirm these findings because *Arsenophonus* sp. was only detected in *A*. *gossypii* in populations where both *A*. *gossypii* and *M*. *persicae* coexisted on the same crop. We report the presence of *Arsenophonus* sp. in populations of *A*. *gossypii* infesting *C*. *annuum* plants (Cayenne var.), which had not been reported by Jousselin *et al*.^44^ in their study.

*Arsenophonus* sp. can have different effects on its hosts, including obligate mutualism in blood-sucking insects^50^, improving the performance of whiteflies^51^, or through facultative mutualism by protecting psyllids against parasitoid attacks^52^. Although the effect of *Arsenophonus* on aphids is still not fully understood, some studies have shown, particularly for the genus *Aphis*, that *Arsenophonus* could be involved in important aspects of aphid ecology. Wagner *et al*.^53^ observed that *Arsenophonus* appears to be involved in the food specialization that the polyphagous aphid *Aphis craccivora* has developed on one of its host plants. Wulff & White^54^ observed that the presence of *Arsenophonus* in individual *A*. *glycines* aphids improved the performance of this insect on aphid-resistant soybean plants.

In addition to the symbionts *B*. *aphidicola* and *Arsenophonus* sp., eight bacterial OTUs were observed at a frequency of 1% or more in at least one sample. After *B*. *aphidicola*, *Pseudomonas* sp. presented the highest frequency in the locality of Dagua (17.48%) and was observed at a frequency of higher than 1% in two additional samples. The OTU of the genus *Pseudomonas* observed in this study was phylogenetically grouped with the species *Pseudomonas fulva* reported in GenBank. *P*. *fulva* has been reported as a symbiotic bacterium in *Hypothenemus hampei* (Coleoptera: Curculionidae), one of the primary pests of coffee. By using caffeine from plants to produce nitrogen, this bacterium allows the coffee borer beetle to survive in coffee plants^55^. Microorganisms of the genus *Pseudomonas*, have also been reported as common members of the bacterial community of insects^41,56^ and it has been suggested that these bacteria have superficial associations with insects, for example, on the surface of the body or, if in the gut, then close to gut orifices. Likewise, *Pseudomonas* and *Sphingomonas*, another genus reported in our study, have been previously described in associations with phloem-feeding insects, in low abundances^17,23,57,58^. Conversely, *Selenomonas*, genus that was found in all of our samples, it is a group of bacteria uncommon in insects. However, this bacterial genus has been reported as part of the intestinal microbiota of the tick, *Amblyomma maculatum*^59^.

Several studies of non-model aphid species have shown that there are bacteria of the family Enterobacteriaceae that have not been described but that may be important in the ecology of aphids^22,43^. For example, Guidolin & Cônsoli^60^ suggest that an unknown genus of this family, which they named Cluster B, can play a key role in helping *Aphis citricidus* to exploit less suitable host plants, complementing the contributions of *B*. *aphidicola*. In our study, the presence of a bacterial OTU belonging to the Enterobacteriaceae family was also reported in all the samples of *A*. *gossypii* and *M*. *persicae*. According to the phylogenetic tree made with sequences of the genebank and genus of known symbionts, this bacterium is closely related to the obligate symbiont *Buchnera*, as well as another genus of the family Enterobacteriaceae reported for the aphid *A*. *glycines*.

The bacterial OTUs found in this study, including the bacterium of the family Enterobacteriaceae, may be playing an important role in the aphid biology that has not yet been reported due to the lack of studies of non-model aphid species. To evaluate this, it is necessary to perform laboratory-based experiments that compare the response of aphids, in terms of performance, in presence or absence of these bacteria, evaluate the location of the symbionts inside the aphids, presence and absence in populations, among others.

By the other hand, there is the possibility that some of these bacteria could be contaminants. Although heritability cannot be discarded, it is likely that this microbes participate in opportunistic associations with these insects, perhaps as gut associates, pathogens, or they could be contaminants from soil, plants or human management. Jousselin *et al*.^61^, report that contaminants accounted for a small proportion of the bacteria identified in their samples, but, the removal of sequences accounting for <1% of the reads in aphid samples would eliminate most of the sequences from contaminants present at low frequency in the aphid samples.

The bacterial communities of *A*. *gossypii* and *M*. *persicae* seem to be structured according to the species of host aphid. The association between the aphid and its bacterial community is affected by a large number of abiotic and biotic factors and may involve the immune system, nutrition, reproduction, communication and many other host systems^62^. According to Henry *et al*.^27^, there is a relationship between the life history traits of the aphids and the cooperative relationships they establish with the symbionts, which may determine the presence of symbionts in certain aphid species or in certain populations within a species. Recent studies have even highlighted that the microbiome is involved in the ability of a given host to transmit a pathogen^63^.

According to our results, geography can be another factor that influence the composition and frequency of bacteria associated with insects^64^. In our study, the locality of Toro showed a microbial community that was differentiated from the other localities and was the only one that presented the endosymbiont *Arsenophonus*. Natural populations of aphids may experience different selection pressures according to geographical location. For example, agricultural management practices^65^, the dynamics of natural enemies and environmental conditions can affect the diversity and frequency of associated bacteria^64^. Tsuchida *et al*.^14^ observed a geographic cline in the distribution and frequency of secondary endosymbionts, in the pea aphid, *A*. *pisum*, which in turn may be related to the host plant species, temperature and precipitation. In the psyllid species *Glycaspis brimblecombei*, significant geographic variation of the endosymbiont *Arsenophonus* was also reported^52^. It is necessary to carry out a greater number of samplings or replicates in each of the localities evaluated in this study to confirm the existing geographical variation, as well as, the prevalence of the *Arsenophonus* symbiont in the locality of Toro.

According to the AMOVA and PCoA results, there are no significant differences between the *A*. *gossypii* microbial communities from the two evaluated pepper species (Cayenne and Tabasco). However, the PCoA shows that the populations of *A*. *gossypii* from the Tabasco and Cayenne varieties in Toro are separated by the composition of their bacterial communities and the symbiont *Arsenophonus* sp. was only detected in the Cayenne variety. In addition, in the Vijes and Toro localities, where both pepper varieties were sampled, and thereby could be compared, simultaneously, it was found the three indices of diversity were higher in the Tabasco variety. These pieces of evidence of possible differences between bacterial communities of Cayenne and Tabasco, can be the starting point for further studies that test the effects of the host plant in aphids.

The bacterial community of *A*. *gossypii* showed fluctuations in diversity and frequency between the different seasons of sampling, suggesting that the microbial community of aphids is dynamic over time. When comparing our results with reports of population dynamics of *A*. *gossypii* by Melo & Manzano^66^, in the same experimental plot of Yotoco, and during the same time period of our study, we observed that only the December sampling, which had the highest bacterial diversity index, correspond with a growth in aphid density as reported by these authors. These results propose that the dynamics of the bacterial community depend on the population dynamics of *A*. *gossypii*. Smith *et al*.^19^ observed that the frequency of bacteria present in aphids can decrease drastically, even over periods as short as three weeks, after seasonal peaks or depending on the frequency of natural enemies. These sudden changes appear to be associated with potentially large costs for aphids, as they have a community of bacteria under certain environmental conditions^21^. Another hypothesis for obtaining greater diversity in one of the samples (December sampling), is the presence of contaminants, especially because there are no replicates of that same season to compare. Diverse studies have shown that bacterial DNA contamination in extraction kits and laboratory reagents can significantly influence the results of microbiota studies, especially samples containing a low microbial load^67^. Among bacterial OTUs reported for the sample taken in December, we found the families Comamonadaceae and Chitinophagaceae reported as possible environmental contaminants^61^ and the family Pelagibacteraceae, reported as commonly present in reagents for the DNA extraction^68^.

This study represents the first approach to the knowledge of the bacterial community present in chili pepper aphids from Colombia. Our results showed that in addition to the primary endosymbiont *B*. *aphidicola*, found in the studied aphids, the secondary symbiont *Arsenophonus* sp, was also found in one population of *A*. *gossypii*. Interesting was finding several genera of bacteria, such as *Pseudomonas* and a genus of the Enterobacteriaceae family, which have not been reported as symbionts. Although differences were found in the bacterial community of *A*. *gossypii* and *M*. *persicae* at the locality, plant and season level, it is possible that these differences are related to contamination events or can be the result of stochastic processes, such as genetic drift, founder effect or isolated cases of symbionts acquisition. Due to this, more in-depth studies, including replicates, are required to confirm whether the differences observed persist both seasonally and in the geographical context.

## Materials and Methods

### Sampling of aphids

Aphid sampling was carried out for crops of *C*. *frutescens* Linneo (Tabasco var.) and *C*. *annum* Linneo (Cayenne var.), located in five localities of southwestern Colombia (Table 1, Fig. 1). Colonies of aphids were collected systematically, taking into account the edges and center of each plot. Each sampling point was separated by at least 20 m to minimize the probability of collecting the offspring of the same mother. To guarantee that the aphids were free of parasitoids, they were collected alive and brought to the laboratory where they were reared under controlled conditions in an environmental chamber (SANYO-MLR-351H) with a photoperiod of 12 hours of light and 12 hours of darkness at a temperature of 25 °C for fifteen days. Captive-born nymphs were excluded from the study. Next, from the colonies of aphids collected in the field, groups of 15 adult apterous surviving aphids were formed from each of the plots sampled (each individual from a different colony) to form a single sample, which was preserved in 96% ethanol at −20 °C until the extraction of DNA. In those localities where *A*. *gossypii* and *M*. *persicae* were observed, 15 aphids of each species were collected. The morphological identification of the aphids was carried out using the key from Blackman & Eastop^13^.

In the locality of Yotoco (Table 1, Fig. 1), the dynamics of the bacterial communities associated with the aphid species *A*. *gossypii* were evaluated on a seasonal basis. For this analysis, four samplings were carried out in an insecticide-free experimental plot between September 2016 and June 2017 with intervals of three months (Table 1). The date and the number of samples taken were chosen considering the average temperature and precipitation recorded by IDEAM (the Institute of Hydrology, Meteorology and Environmental Studies) for 2014 and 2015 (http://www.ideam.gov.co/web/tiempo-y-clima/climatologico-mensual, visited in November 2016). The two peaks of maximum precipitation and minimum temperature occurred during the months of March-April and October-November, and the two peaks of minimum precipitation and maximum temperature occurred during the months of December-January and June-July.

### DNA extraction, amplification and sequencing of the 16S rRNA gene

Prior to DNA extraction, aphid samples were washed for 5 min in 70% ethanol and rinsed three times with sterile water to remove surface contamination. DNA was extracted from surface sterilized aphids (15 per sample) using a commercial DNeasy® Blood & Tissue kit (QIAGEN) kit, following the manufacturer’s protocol. The amplification of the 16S rRNA gene was carried out using the universal primers Bakt_341F (CCTACGGGNGGCWGCAG) and Bakt_805R (GACTACHVGGGTATCTAATCC)^69^, which amplify the V3 and V4 hypervariable regions. The sequencing was obtained on a MiSeq Illumina platform generating 300-bp paired reads. Additionally, the NCBI Primer-BLAST tool was used to verify that the primers sequences were present on the genome of the most common symbionts in aphids (*S*. *symbiotica*, *H*. *defensa* and *R*. *insecticola*). The entire amplification and sequencing process was performed by Macrogen Corp. (http://support.illumina.com/downloads/16s_metagenomic_sequencing_library_preparation.html).

### Data analysis

#### Sequence analysis and taxonomic assignments

Twenty-thousand sequences per sample were processed using the program *Mothur* (v 1.39.5)^70^ (http://www.mothur.org/) according to the protocol described in the *MiSeq Standard Operating Procedure*^71,72^. The maximum length of the allowed sequences was 465 bp. We used the *Uchime* algorithm^73^, implemented in *Mothur* to detect chimeric sequences (sequences resulting from the recombination of two different taxa sequences due to jumping events in the PCR). This procedure was carried out for each sample and the identified chimeric sequences were excluded from the data set. The operational taxonomic units (OTUs) were defined at a level of 97% similarity, and a taxonomic assignment was subsequently made using the *Greengenes reference taxonomy* database as a reference and a minimum confidence value of 0.8 (https://www.mothur.org/wiki/Greengenes-formatted_databases).

#### Phylogenetic reconstruction

Representative sequences of OTUs with frequencies equal to or greater than 1% in the different samples were selected, as this threshold allowed us to reduce the probability of processing sequences from contaminating bacteria^61^. Subsequently, these sequences were used to search for symbiotic and free-living bacteria at the databases using the BLASTn tool at NCBI web interface and the database Nucleotides Collection (nr/nt). A phylogenetic analysis of the whole set of sequences was carried out using the maximum likelihood method, which also included GenBank sequences. The alignment was made using the ClustalW algorithm^74^ in MEGA 7^75^. Phylogenetic reconstruction was achieved using the RAxML-HPC BlackBox program (8.2.8)^76^ through the CIPRES platform^77^ with automatic bootstrapping and the other criteria applied by default.

#### Analysis of bacterial communities among aphids, localities and host plants

From the taxonomic allocation file and their respective absolute frequencies, the relative frequencies were calculated and the OTUs were selected with a representation of at least 1% in some of the samples. Rarefaction curves were generated and diversity descriptors were calculated, including Simpson’s index, the Shannon index and the Chao index using *Mothur*, to quantify the diversity and dominance of bacteria by aphid species, locality, host plant and season. To explore differences in the structure of the bacterial communities between the aphid species and between the host plants (in the latter comparison, only the *A*. *gossypii* samples were included, since they have greater representation in both host plants), a PCoA was realized using *Mothur* with the weighted (includes relative abundance of each OTU) and unweighted (only absence or presence of each OTU) UniFrac distances. UniFrac is a measure of distance that is used to calculate differences between bacterial communities based on phylogenetic information^78^. Finally, an AMOVA^69,78–81^ was carried out in *Mothur* to evaluate if there were significant differences between the bacterial communities of the *A*. *gossypii* aphid, taking into account the host plant.

## Supplementary information

Dataset 1
