# Supplementary material for: Bacterial communities of Aphis gossypii and Myzus persicae (Hemiptera: Aphididae) from pepper crops (Capsicum sp.)
Source: Sci Rep. 2019 Apr 8;9:5766. doi: 10.1038/s41598-019-42232-8 (PMC6453963; doi:10.1038/s41598-019-42232-8)
Supplement: Supplementary file 1 — Dataset 1 [file 41598_2019_42232_MOESM1_ESM.docx]

**Supplementary information**

**Bacterial communities of *Aphis gossypii* and *Myzus persicae* (Hemiptera: Aphididae) from pepper crops (*Capsicum* sp).**

**Jenny Johana Gallo-Franco^1,2^*, Diana Nataly Duque-Gamboa^1,2^ & Nelson Toro-Perea^1,2^.**

^1^Biology department (Departamento de biología), Universidad del Valle, Street 13 No. 100-00, 760032, Cali, Colombia.

^2^Centre for Bioinformatics and Photonics-CIBioFi, Universidad del Valle, Street 13 # 100-00, Building 320 No. 4076, 760032, Cali, Colombia

*Corresponding email: jenny.gallo@correounivalle.edu.co

**This Supplementary Information contains:**

Supplementary Figure Legends

Supplementary Figures S1-S6

**Supplementary Figure Legends**

**Figure S1.** Rarefaction curves based on the number of OTUs present in each sample taking into account a dissimilarity level of 3%. Sampling localities: Vijes (V), Dagua (D), Bolivar (B), Toro (T). Aphid host plants: Tabasco (1), Cayenne (2). Aphid species: A. gossypii (Ag), M. persicae (Mp).

**Figure S2.** Relative abundance of bacteria at phylum level of A. gossypii and M. persicae in pepper crops. Sampling localities: Vijes (V), Dagua (D), Bolivar (B), Toro (T). Aphid host plants: Tabasco (1), Cayenne (2). Aphid species: A. gossypii (Ag), M. persicae (Mp).

**Figure S3**. Relative abundance of bacteria at Class level of A. gossypii and M. persicae in pepper crops. Sampling localities: Vijes (V), Dagua (D), Bolivar (B), Toro (T). Aphid host plants: Tabasco (1), Cayenne (2). Aphid species: A. gossypii (Ag), M. persicae (Mp).

**Figure S4.** Relative abundance of bacteria at family level for A. gossypii and M. persicae in pepper crops.

**Figure S5**. Rarefaction curves based on the number of OTUs present in four each different times point of the year taking into account a dissimilarity level of 3%. Aphid species: A. gossypii (Ag). Time points: September of 2016 (Sep_16), December of 2016 (Dic_16), March of 2017 (Mar_17), June of 2017 (Jun_17).

**Figure S6.** Relative abundance of bacteria at the phylum level of A. gossypii in the experimental plot of the Yotoco locality. September of 2016 (Sep_16), December of 2016 (Dic_16), March of 2017 (Mar_17), June of 2017 (Jun_17).


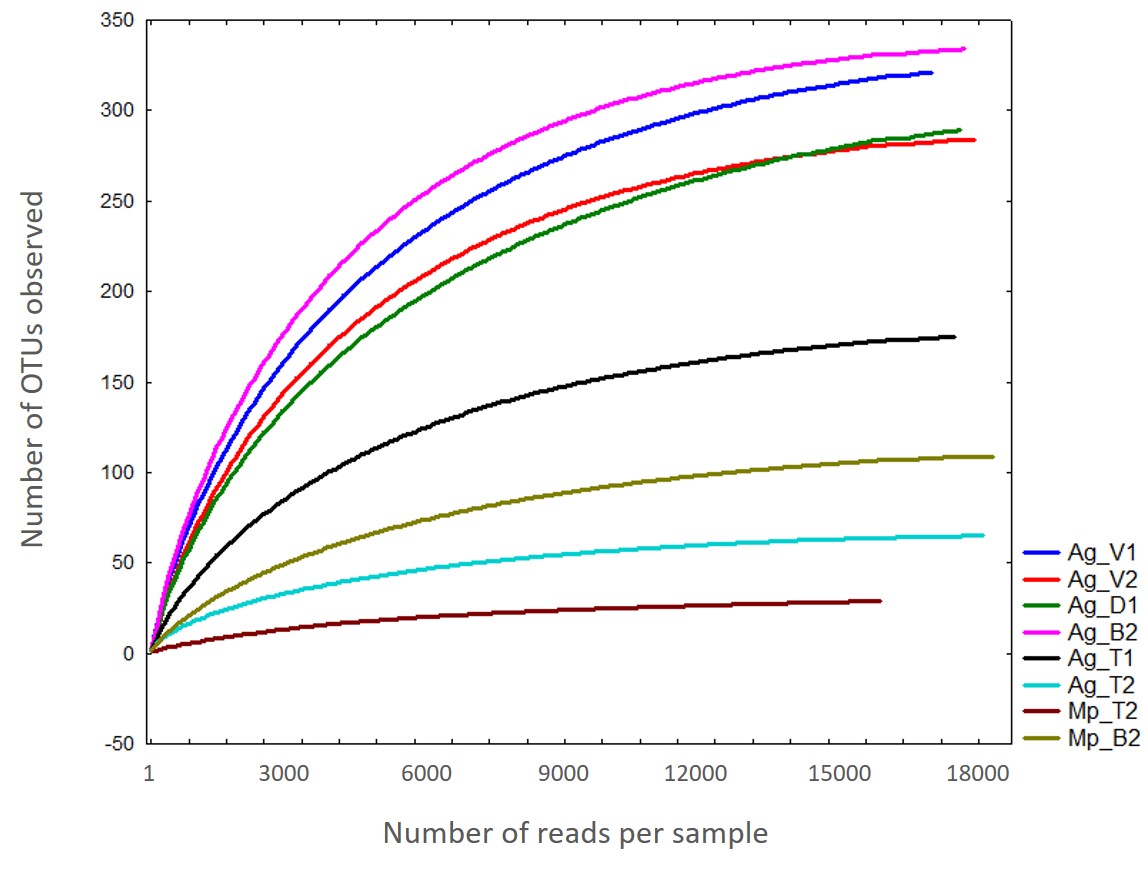


**Figure S1**

**Figure S2**

**Figure S3**

**Figure S4**


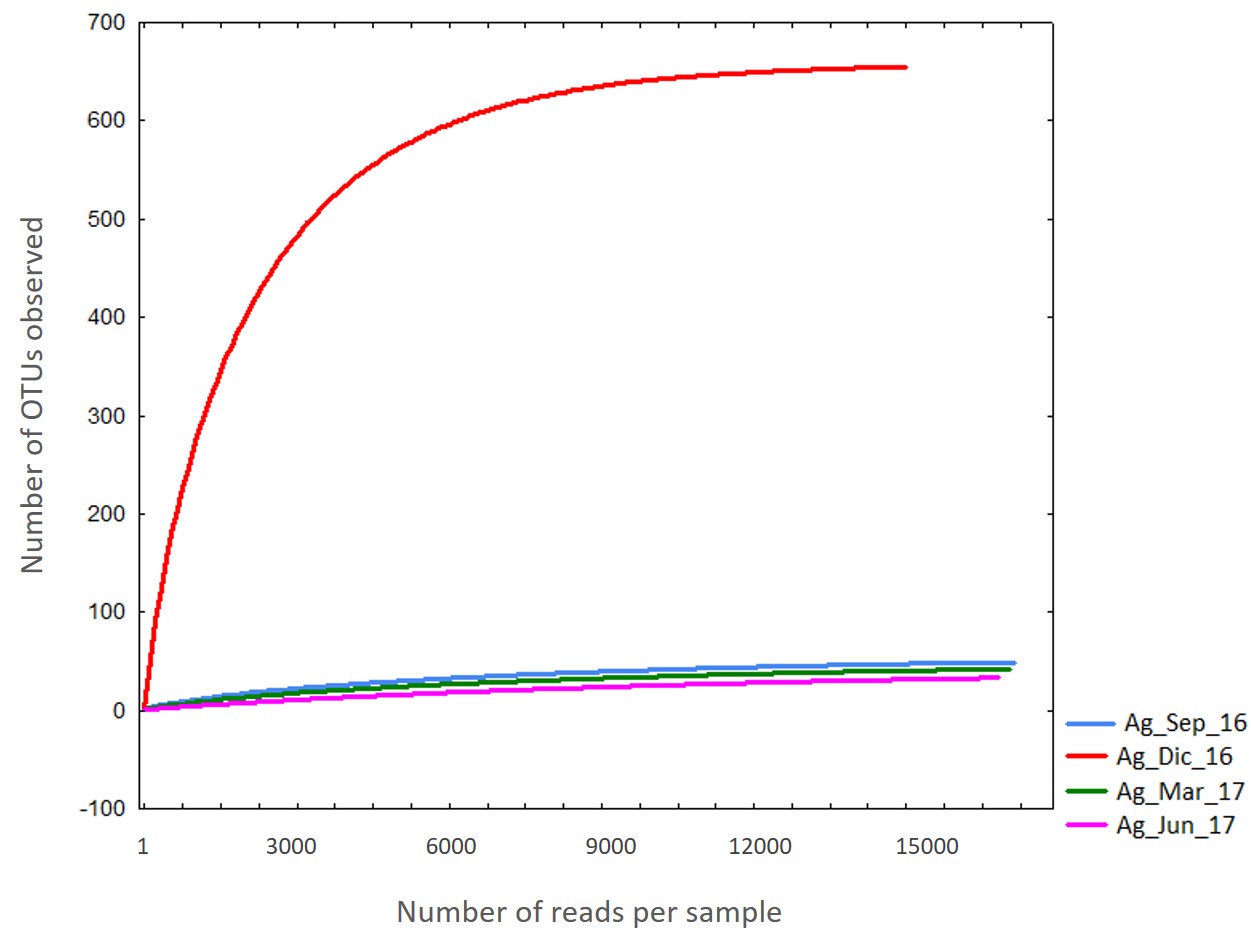


**Figure S5**

**Figure S6**
